# Knockdown of glucose-regulated protein 78 enhances poly(ADP-ribose) polymerase cleavage in human pancreatic cancer cells exposed to endoplasmic reticulum stress

**DOI:** 10.3892/or.2014.3533

**Published:** 2014-10-06

**Authors:** XIA JIANG, TATSUO KANDA, SHINGO NAKAMOTO, YUKI HAGA, REINA SASAKI, MASATO NAKAMURA, SHUANG WU, RINTARO MIKATA, OSAMU YOKOSUKA

**Affiliations:** 1 Department of Gastroenterology and Nephrology, Chiba University, Graduate School of Medicine, Chuo-ku, Chiba 260-8677, Japan; 2 Department of Molecular Virology, Chiba University, Graduate School of Medicine, Chuo-ku, Chiba 260-8677, Japan

**Keywords:** apoptosis, ER stress, GRP78/Bip, pancreatic cancer, resistance, UPR

## Abstract

The present study examined the expression of glucose-regulated protein 78 (GRP78/Bip) in human pancreatic cancer cell lines and the effect of knockdown of GRP78 on the cleavage of poly(ADP-ribose) polymerase (PARP). Human pancreatic cancer cell lines (KP-2, MIAPaCa-2, Panc-1 and SUIT-2), constitutively expressed GRP78. We also demonstrated that ER stress induced by thapsigargin upregulated protein levels of GRP78. In the presence of thapsigargin, knockdown of GRP78 enhanced the PARP cleavage in the human pancreatic cancer cells. These results provide evidence that GRP78 is a potential therapeutic target for ‘difficult-to-treat’ pancreatic cancer, in which ER stress signaling in part falls into disorder.

## Introduction

Pancreatic cancer is almost the deadliest of all malignancies (1). In Japan, pancreatic cancer is currently the fifth leading cause of cancer-related death among individuals of both genders (2,3). Resection surgery is still the only potentially curative treatment for pancreatic cancer, and recent improvements in operative technique have been reported (4). Although advances in adjuvant treatment have been observed (5), in general, the prognosis of patients with pancreatic cancer is still poor. Further studies of the mechanisms of pancreatic carcinogenesis and cancer development are needed, and new therapeutic options are highly desirable.

Endoplasmic reticulum (ER) stress response in tumor cells is critical for tumor cell growth and cancer progression (6). The ER stress response is mediated by at least three sensor molecules: inositol-requiring enzyme 1α (IRE1α), PKR-like ER kinase (PERK), and activating transcription factor 6 (ATF6), which are usually associated with glucose-regulated protein 78 (GRP78/Bip) (7). ER stress, which is associated with the accumulation of unfolded proteins, induces unfolded protein response (UPR), yet if ER stress is overloaded, cells could face death such as by apoptosis and autophagy. Downstream of IRE1α and PERK, the effector molecules, X-box-binding protein 1 (XBP1) and C/EBP homologous protein (CHOP), and growth arrest and DNA damage gene 34 (GADD34) all exist, and they are activated by ER stress. ER stress also leads to the phosphorylation of eukaryotic translation initiation factor 2α (eIF2α) (8). For example, p90ATF6 is converted to the activated form p50ATF6, and p50ATF6 translocates to the nucleus (9). Basic leucine-zipper family factors p50ATF6 and XBP1 could induce expression of a subset of UPR-related genes, which include ER stress elements, and are involved in efficient protein folding, maturation and degradation in the ER (6).

The association between ER stress response and tumor growth and progression has been reported (10). We and others have reported that GRP78 is involved in cancer development and innate immune response in the liver (11–14). Liver and pancreas progenitors commonly develop from endoderm cells in the embryonic foregut (15). Pancreatic epithelial cells have a highly developed ER due to a strong engagement in digestive enzyme secretion (16). GRP78 is the main target of UPR signaling that promotes pancreatic cancer cell survival (17). GRP78 is involved in cancer progression as well as drug resistance (18,19). Hence, to decrease the ability of pancreatic cancer cells to survive and proliferate, it may be necessary to block GRP78 expression (17).

We previously demonstrated that blocking of the induction of UPR, as well as inhibition of GRP78 expression is associated with the cleavage of poly(ADP-ribose) polymerase (PARP) (13). In the present study, we examined the expression of ER stress-related molecules in human pancreatic cancer cell lines in the presence or absence of thapsigargin, one of the ER stress-inducers. We also investigated whether knockdown of GRP78 by small interfering RNA (siRNA) enhances the PARP cleavage in human pancreatic cancer cell lines exposed to ER stress.

## Materials and methods

### Cell culture

Human pancreatic cancer cell lines (KP-2, MIAPaCa-2, Panc-1 and SUIT-2) were grown in RPMI-1640 medium (Sigma, St. Louis, MO, USA) supplemented with 10% fetal bovine serum, 100 U/ml penicillin and 100 μg/ml streptomycin at 37°C in a humidified atmosphere with 5% CO_2_. Inhibitor of sarcoplasmic/endoplasmic reticulum (ER) Ca^2+^ ATPases (SERCA), thapsigargin, control siRNA (si-control) and siRNA for GRP78 (si-GRP78) were purchased from BioVision (Milpitas, CA, USA) and Santa Cruz Biotechnology (Santa Cruz, CA, USA), respectively.

### Western blotting

Twenty-four hours after thapsigargin (1 μM) treatment, cells were lysed in sodium dodecyl sulfate sample buffer, and after sonication, lysates were processed for western blot analysis (11). Briefly, protein samples were subjected to electrophoresis on 5–20% polyacrylamide gels and transferred onto polyvinylidene difluoride membranes (ATTO, Tokyo, Japan). Membranes were probed with antibodies specific for ATF4, ATF6 and tubulin (Abcam, Cambridge, UK); GADD34, gyceraldehyde-3-phosphate dehydrogenase (GAPDH) and XBP1 (Santa Cruz); eIF2α, phospho-eIF2α (Ser51), GRP78/Bip and PARP (Cell Signaling Technology, Tokyo, Japan). After washing with PBS-T, the membranes were incubated with secondary horseradish peroxidase-conjugated antibodies. Signals were detected by means of enhanced chemiluminescence (GE Healthcare, Tokyo, Japan) and scanned by image analyzer LAS-4000 and Image Gauge (version 3.1) (Fuji Film, Tokyo, Japan) and ImageJ software (NIH, Bethesda, MD, USA).

### Transfection of siRNA

To confirm the effects of GRP78 knockdown on apoptosis, we examined GRP78 knockdown by small-interfering RNA (siRNA). Cells were transfected with 50 nM si-GRP78 or si-control, using Effectene transfection reagent (Qiagen, Hilden, Germany) according to the manufacturer’s protocol (20). After 24 h of transfection, cells were treated with 1 μM thapsigargin for 24 h.

### Statistical analysis

Results are expressed as means ± standard deviation (SD). Statistical analysis was performed using the Student’s t-test. A P-value <0.05 was considered to indicate a statistically significant result.

## Results

### Human pancreatic cancer cell lines express GRP78

**Figure 1:**
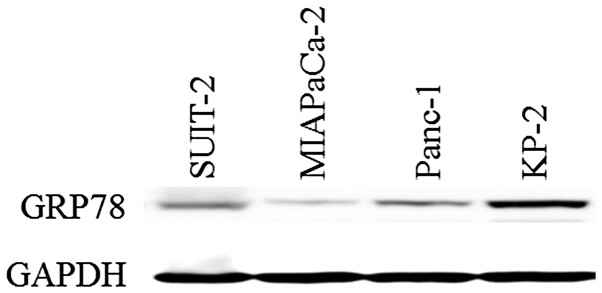
Glucose-regulated protein 78 (GRP78/Bip) is expressed at various levels in the human pancreatic cancer cell lines. Western blot analyses of GRP78 and glyceraldehydes 3-phosphate dehydrogenase (GAPDH) in SUIT-2, MIAPaCa-2, Panc-1 and KP-2 cells.

First, we examined the GRP78 expression in the human pancreatic cancer cell lines SUIT-2, MIAPaCa-2, Panc-1 and KP-2 (3). Protein samples were collected from the four pancreatic cancer cell lines, and protein levels of GRP78 were investigated by western blotting with a specific antibody for GRP78 (Fig. 1). We confirmed that all four pancreatic cancer cell lines variably expressed GRP78.

### Thapsigargin upregulates the protein levels of GRP78 in the human pancreatic cancer cell lines

**Figure 2:**
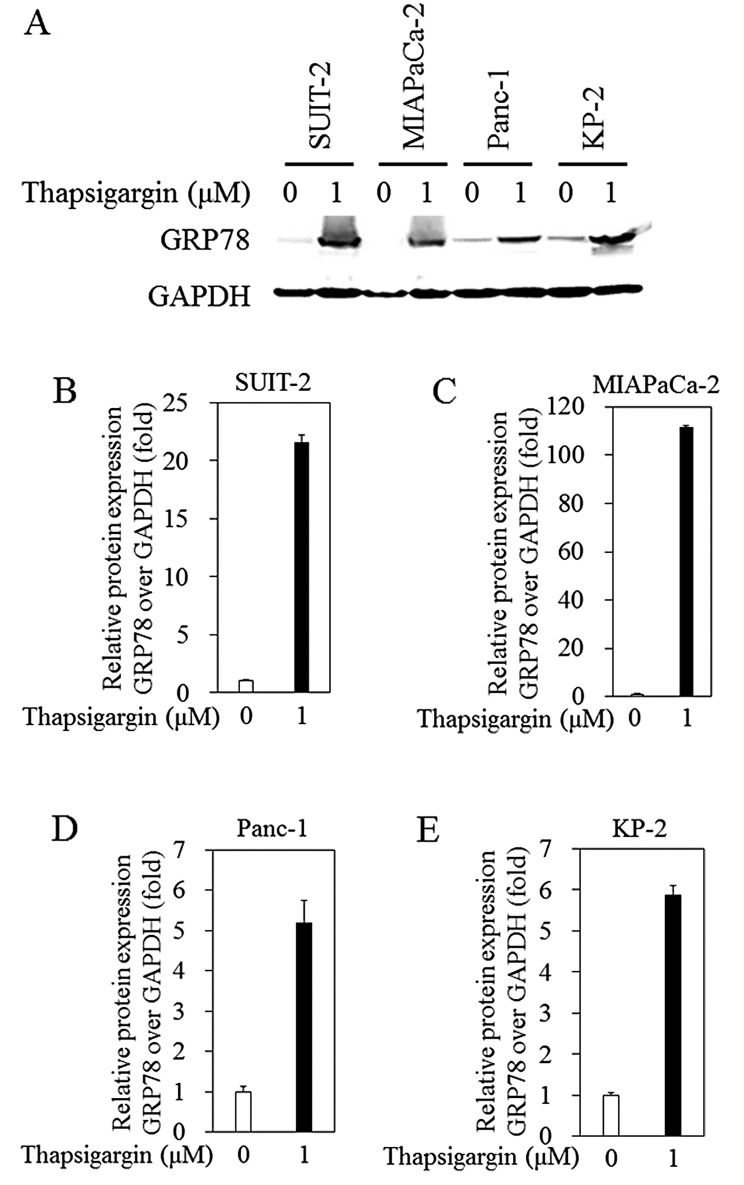
Effects of thapsigargin on glucose-regulated protein 78 (GRP78/Bip) expression in human pancreatic cancer cell lines. (A) Western blot analyses of GRP78 and GAPDH in SUIT-2, MIAPaCa-2, Panc-1 and KP-2 cells treated with or without 1 μM thapsigargin for 24 h. GRP78/glyceraldehyde 3-phosphate dehydrogenase (GAPDH) ratios from 3 independent experiments were measured using ImageJ software in (B) SUIT-2, (C) MIAPaCa-2, (D) Panc-1 and (E) KP-2 cells, respectively.

Next, we examined the effect of thapsigargin, one of the ER stress-inducers, on GRP78 expression in the human pancreatic cancer cell lines (Fig. 2). Treatment of 1 μM thapsigargin for 24 h led to the upregulation of GRP78 expression at the protein level [21.5±0.7 vs. 1±0.1 (in untreated control), n=3, p=0.00015; 111.5±1.0 vs. 1±0.12, n=3, p=0.000010; 5.2±0.57 vs. 1±0.1, n=3, p=0.0023; and 5.9±0.2 vs. 1±0.1, n=3, p=0.00013, respectively, in the SUIT-2, MIAPaCa-2, Panc-1 and KP-2 cells]. In the MIAPaCa-2, cells GRP78 expression was more strongly induced than in the other three cell lines.

### Effects of thapsigargin on GADD34, ATF4, ATF6 and XBP1 protein expression levels in the human pancreatic cancer cell lines

**Figure 3:**
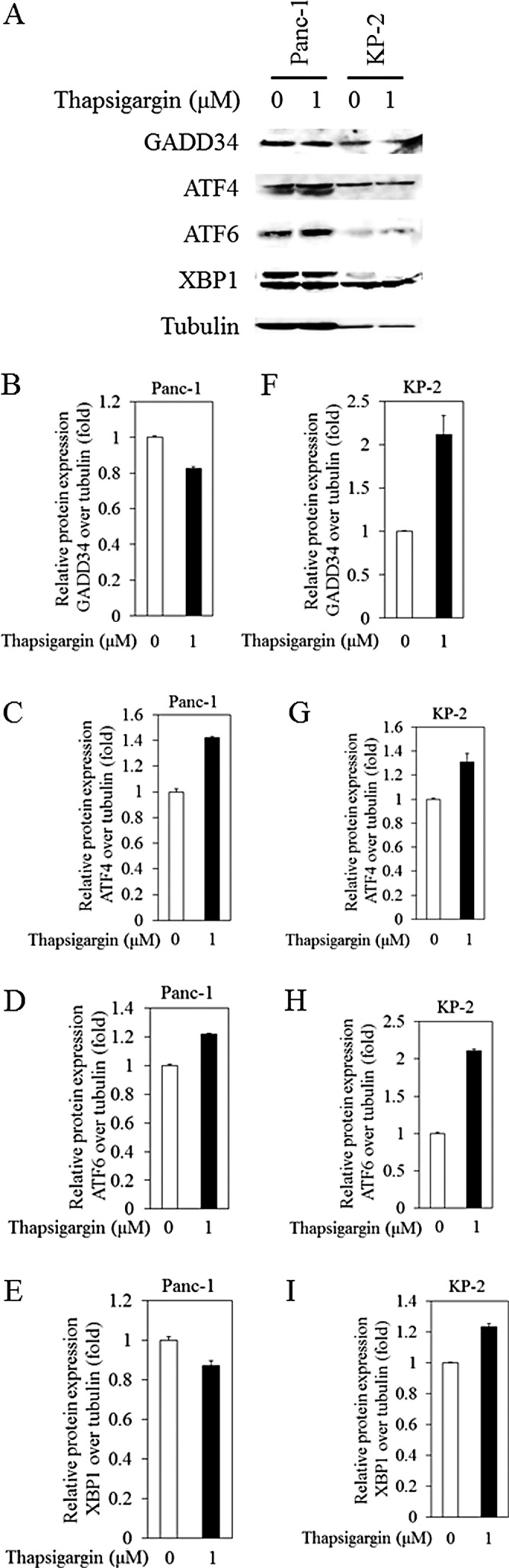
Effects of thapsigargin on growth arrest and DNA damage gene 34 (GADD34), activating transcription factor 4 (ATF4), ATF6 and X-box-binding protein 1 (XBP1) expression in human pancreatic cancer cell lines Panc-1 and KP-2. (A) Western blot analyses of GADD34, ATF4, ATF6, XBP1 and tubulin in Panc-1 and KP-2 cells treated with or without 1 μM thapsigargin for 24 h. (B) The ratios of GADD34 over tubulin, (C) ATF4 over tubulin, (D) ATF6 over tubulin and (E) XBP1 over tubulin in Panc-1 cells were measured using ImageJ software. (F) The ratios of GADD34 over tubulin, (G) ATF4 over tubulin, (H) ATF6 over tubulin and (I) XBP1 over tubulin in KP-2 cells were also measured using ImageJ software.

We examined the protein expression of ER stress signaling-associated molecules in the human pancreatic cell lines treated with or without thapsigargin. The results for the Panc-1 and KP-2 cells are shown in Fig. 3. In the Panc-1 cells, ATF4 and ATF6 expression was upregulated in the presence of 1 μM thapsigargin [1.4±0.010 vs. 1±0.023 (in untreated control), n=3, p=0.000089; and 1.2±0.0027 vs. 1±0.010, n=3, p=0.00019, respectively] (Fig. 3A, C and D. In the Panc-1 cells, GADD34 and XBP1 expression at the protein level was down-regulated in the presence of 1 μM thapsigargin [0.82±0.012 vs. 1±0.0076 (in untreated control), n=3, p=0.0000414; and 0.87±0.024 vs. 1±0.019, n=3, p=0.0012, respectively] (Fig. 3A, B and E).

On the other hand, in KP-2 cells, the protein expression levels of GADD34, ATF4, ATF6 and XBP1 were upregulated in the presence of 1 μM thapsigargin [2.1±0.22 vs. 1±0.012 (in untreated control), n=3, p=0.0063; 1.3±0.073 vs. 1±0.0062, n=3, p=0.0088; 2.1±0.022 vs. 1±0.014, n=3, p=0.0000008; and 1.2±0.019 vs. 1±0.0063, n=3, p=0.00043, respectively] (Fig. 3A and F–I).

XBP1 was also upregulated in the presence of 1 μM thapsigargin in both SUIT-2 and MIAPaCa-2 cells, yet we did not observe any enhancement of GADD34, ATF4 or ATF6 by thapsigargin (data not shown).

### Effects of thapsigargin on the phosphorylation of eIF2α in the human pancreatic cancer cell lines

**Figure 4:**
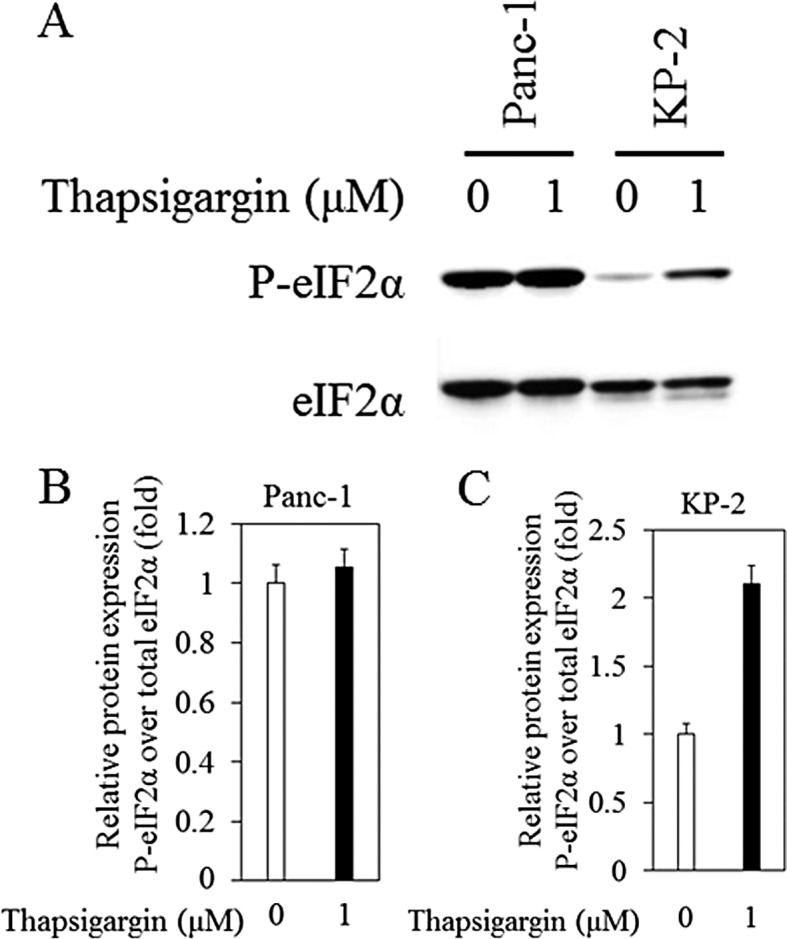
Effects of thapsigargin on the phosphorylation of Ser51-eIF2α (p-eIF2α) in human pancreatic cancer cell lines Panc-1 and KP-2. (A) Western blot analyses of p-eIF2α and total eIF2α in Panc-1 and KP-2 cells treated with or without 1 μM thapsigargin for 24 h. The ratios of p-eIF2α over total eIF2α were measured using ImageJ software in (B) Panc-1 and (C) KP-2 cells.

We also examined the phosphorylation status of eIF2α to understand how thapsigargin affects ER stress signaling in Panc-1 and KP-2 cells (Fig. 4A). In Panc-1 cells, phosphorylation of Ser51-eIF2α in the presence of thapsigargin tended to increase, compared with that in the absence of thapsigargin (Fig. 4B; 1.1±0.059 vs. 1±0.064, n=3, p=0.17). In the KP-2 cells, significant phosphorylation of Ser51-eIF2α in the presence of thapsigargin was observed when compared with that in the absence of thapsigargin (Fig. 4C; 2.1±0.14 vs. 1±0.075, n=3, p=0.00050).

### Knockdown of endogenous GRP78 enhances PARP cleavage in the pancreatic cancer cells

**Figure 5:**
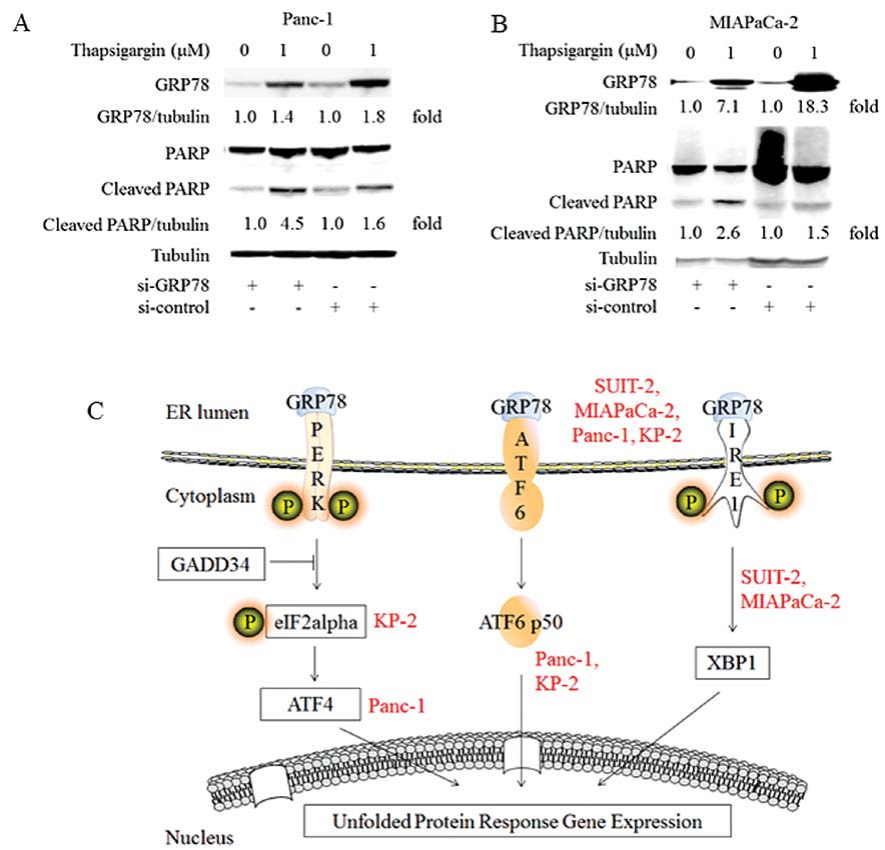
Knockdown of endogenous glucose-regulated protein 78 (GRP78/Bip) by siRNA enhances poly(ADP-ribose) polymerase (PARP) cleavage in pancreatic cancer cells. Western blot analyses of GRP78 and tubulin in (A) Panc-1 and (B) MIAPaCa-2 cells treated with or without 1 μM thapsigargin for 24 h. Cell lysates were analyzed for GRP78, PARP and tubulin expression using specific antibodies. Bands were analyzed using ImageJ software. (C) Schematic presentation of endoplasmic reticulum stress (ER) pathways in human pancreatic cancer cell lines.

We confirmed that the expression of GRP78 at the protein level was upregulated in all four human pancreatic cancer cell lines tested, yet other molecules downstream of GRP78 reported to be involved in ER stress were expressed at variable levels depending on the individual cell line. Thus, we focused our examination on GRP78. Our previous study (13) demonstrated that blocking of GRP78 induction led to PARP cleavage in hepatocyte apoptosis. We investigated the effect of knockdown of GRP78 by siRNA on PARP cleavage in pancreatic cancer cells treated with thapsigargin (Fig. 5A and B).

GRP78 expression was significantly inhibited by transfection with si-GRP78 in the presence of thapsigargin, compared with that with si-control [1.4±0.040 vs. 1.8±0.040, n=3, p=0.00014; and 7.1±0.24 vs. 18.3±0.37, n=3, p=0.0000038, respectively, in Panc-1 (Fig. 5A) and MIAPaCa-2 cells (Fig. 5B)].

PARP cleavage was significantly enhanced by transfection with si-GRP78 in the presence of thapsigargin, compared with that with si-control [4.5±0.045 vs. 1.6±0.085, n=3, p=0.00000080; and 2.6±0.13 vs. 1.5±0.047, n=3, p=0.00016, respectively, in Panc-1 (Fig. 5A) and MIAPaCa-2 cells (Fig. 5B)].

## Discussion

In the present study, we demonstrated that i) human pancreatic cancer cell lines expressed GRP78; ii) ER stress induced by thapsigargin upregulated protein levels of GRP78 in human pancreatic cancer cell lines; iii) ER stress-related molecules downstream of GRP78 were expressed at various levels according to the respective human pancreatic cancer cell lines; and iv) finally, knockdown of GRP78 by siRNA enhanced PARP cleavage in the human pancreatic cancer cell lines. To our knowledge, this is the first report to show the association between GRP78 and PARP cleavage in pancreatic cancer cell lines treated with thapsigargin.

Our results that human pancreatic cancer cell lines express GRP78 supported a previous study (21) showing that the heat shock proteins HSP90 and GRP78 are constitutively expressed in gastrointestinal cancers including human pancreatic cancer. We also observed that ER stress induced by thapsigargin upregulated protein levels of GRP78 in human pancreatic cancer cell lines. However, ER stress-related molecules downstream of GRP78, such as GADD34, ATF4, ATF6, XBP1 and phospho-eIF2α were not constitutively increased by thapsigargin, but rather were dependent on individual cell lines (Figs. 2–4). These results suggest that GRP78 may have an impact on many different cellular processes and survival of pancreatic cancer and that ER stress signaling downstream of GRP78 can be expected to be disturbed in pancreatic cancer.

It was reported that an increase in GRP78 expression in pancreatic cancer cells may enhance and account for the altered sensitivity of pancreatic cancer to chemotherapeutic agents (21). UPR regulator GRP78 is an anti-apoptotic protein that is usually upregulated in cancer and plays a critical role in chemoresistance in various types of cancers (22). Recently it was also reported that UPR induction in tumor endothelial cells under an acidic pH condition is related to chemoresistance and may contribute to therapeutic failure in response to chemotherapy (23). It was also reported that GRP78 is overexpressed in malignant cells resistant to therapy (24).

PARP is one of the proteins processed by post-translational modification and plays a crucial role in many processes, including DNA repair and cell death (25). During apoptosis, caspases cause PARP cleavage and inactivation, in which PARP proteolysis produces an 89-kDa C-terminal fragment and a 24-kDa N-terminal (25). We observed that in the presence of thapsigargin, knockdown of GRP78 enhanced PARP cleavage in human pancreatic cancer cells Panc-1 as well as MIAPaCa-2. Wang *et al* reported that suppression of GRP78 by taxol and vinblastine potentiated the activation of JNK phosphorylation, caspase-7 and PARP cleavage in the human breast cancer cell line MCF-7 (26). The Hsp90 inhibitor SNX-2112 also induced PARP cleavage as well as the reduction in GRP78 expression in the multidrug-resistant human chronic myeloid leukemia K562/ADR cell line (27).

Collectively, our results suggest that both GRP78 and PARP may have key roles in the chemoresistance of pancreatic cancer (28) and that GRP78 may be one of the valid targets against chemoresistance (24). In conclusion, GRP78 is a potential therapeutic target for ‘difficult-to-treat’ pancreatic cancer, in which ER stress signaling in part falls into disorder.
